# Supplementary material for: Psychological capital and social class: A capital approach to understanding positive psychological states and their role in explaining social inequalities
Source: PLoS One. 2024 Sep 9;19(9):e0310031. doi: 10.1371/journal.pone.0310031 (PMC11383244; doi:10.1371/journal.pone.0310031)
Supplement: S1 Text — (DOCX) [file pone.0310031.s001.docx]

**S1 Text. Structural models.**


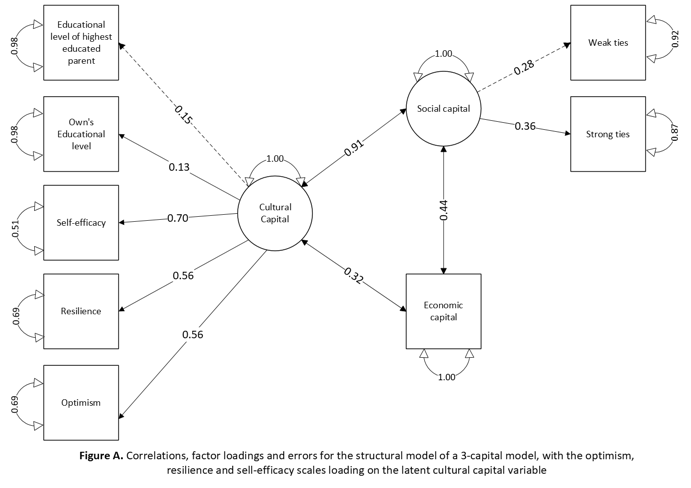


**Fig A. Correlations, factor loadings and errors for the structural model of a 3-capital model, with optimism, resilience and self-efficacy scales loading on the latent cultural capital variable.**


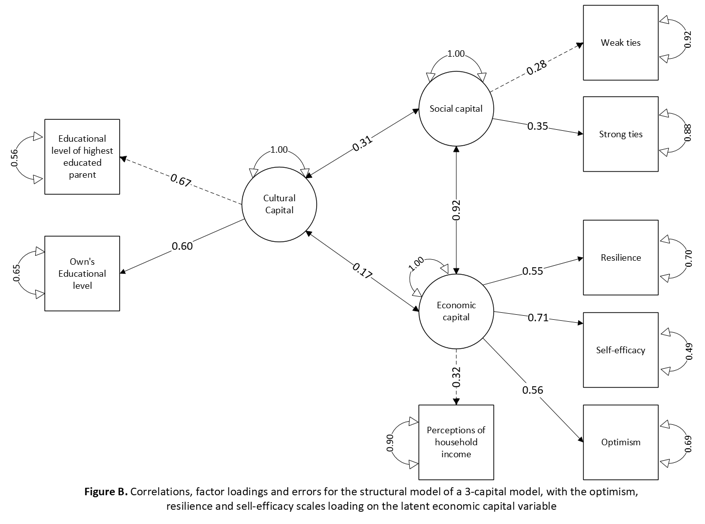


**Fig B. Correlations, factor loadings and errors for the structural model of a 3-capital model, with optimism, resilience and self-efficacy scales loading on the latent economic capital variable.**


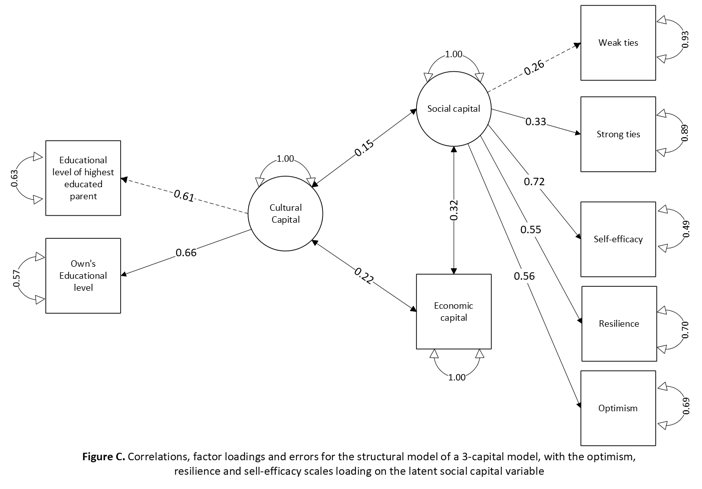


**Fig C. Correlations, factor loadings and errors for the structural model of a 3-capital model, with optimism, resilience and self-efficacy scales loading on the latent social capital variable.**


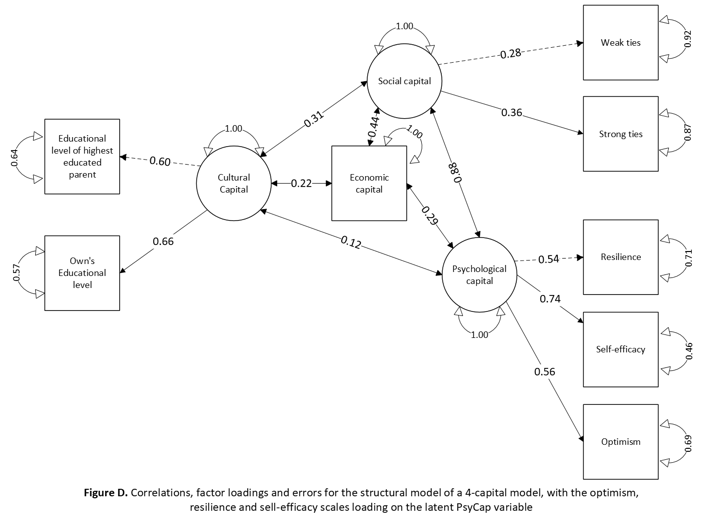
**Fig D. Correlations, factor loadings and errors for the structural model of a 4-capital model, with optimism, resilience and self-efficacy scales loading on the latent PsyCap variable.**
