# Supplementary material for: Psychological capital and social class: A capital approach to understanding positive psychological states and their role in explaining social inequalities
Source: PLoS One. 2024 Sep 9;19(9):e0310031. doi: 10.1371/journal.pone.0310031 (PMC11383244; doi:10.1371/journal.pone.0310031)
Supplement: Attachment — Submitted filename: Response to Reviewers.docx [file pone.0310031.s002.docx]

**Reviewer #1:**

[Authors]: Dear Reviewer #1, we want to explicitly thank you for taking the time to review our revised manuscript with great care. We appreciate your insights and are confident that the manuscript only improved by adjusting the manuscript in accordance with your feedback.

1) The authors response to comment 3 is not completely satisfying. Ridit scores do not remove country-level effects, but make the measures more comparable over countries. For instance, what is considered high wealth or highly educated can differ between countries. When calculating ridit scores for each measure and per country separately, before placing individuals into a low, middle, or high group**,** makes measures more comparable over countries. In this case you are not comparing oranges with apples. Calculating ridit scores is therefore especially important when using more objective measures. On the other hand, I do not think the results will change much if the authors would have included ridit scores, since they mostly recoded their measures into quite crude groups (categorized indicators into 3 groups (low-mean-high)) and mostly also did not use objective measures but more subjective measures, where one could expect that these should be comparable over countries.

However, it might be good to have a statistician have a look, since the sophisticated methods the authors used go beyond my own knowledge.

[Authors]: Thank you for pointing our attention to this important discussion. The discussion about the usage of ridit-scores touches upon a fundamental debate regarding comparing social classes across different countries. This debate revolves around whether we should focus on absolute differences or relative differences. We agree with Reviewer 1 that relative differences matter. To some extent, when comparing personal or household income across countries, relative income comparisons allow for a more accurate assessment of the standard of living within and between countries. Comparing income across countries in a relative way takes into account differences in, for example, purchasing power. A certain income level might afford a higher standard of living in a country with a lower cost of living compared to a country with a higher cost of living. By considering relative income, we could get a more accurate picture of what individuals can afford with their income in different countries. So, ridit scores could provide insights into how income levels in one country compare to those in others, taking into account differences in purchasing power and cost of living between countries.

Yet, for household income (or economic capital in our manuscript), we already using a subjective measure of income: *perception of the current household income with answer categories: (1) Living comfortably on present income; (2) Coping on present income; (3) Difficult on present income and (4) Very difficult on present income. The consequence is that we already take country-specific issues into account that could impact the “purchasing power” of an income.*

The same goes for psychological capital. People evaluate themselves and their circumstances relative to others. Psychological measures that capture constructs like self-esteem, and resilience are influenced by comparisons with others (see, for instance, social comparison theory). Yet, here too, the different sub-questions that make up the PsyCap scale are subjective questions. For instance, “In general I feel very positive about myself”, with answer categories ranging from 1 (agree strongly) to 5 (disagree strongly). By using these subjective questions, *we automatically take country-specific issues into account that could impact how high or low a person might evaluate his or her PsyCap.*

*The same reasonings can be made for the variable “strong ties” and “weak ties” of social capital, as this is also measured using a subjective measure.*

However, regarding cultural capital, we are using respondents’ own and their parent’s educational attainment based on the ISCED scale. This is measured in an “absolute” manner. Yet, the International Standard Classification of Education (ISCED) exists precisely because we recognize and categorize educational levels in an absolute sense, irrespective of relative differences between countries. *Therefore, we are confident in the presentation of our results.*

2) Regarding the third aim of the paper, where the authors included the suggested aim of examining country differences in the distribution of social classes, it would have been nice if the authors:

**a. included hypotheses into** the objectives section of the introduction, about the specific country-level indicators they are expecting results.

[Authors]: Thank you for this suggestion, we agree with Reviewer 1 that including hypothesis will improve the manuscript. We included two hypotheses based on the research of Andersen & Curtis (2012) and Breen (2010):

Our last aim (objective 3) is to examine cross-national differences in social classes distribution. The sizes of social classes within European countries are influenced by macro-level factors, such as a country’s level of economic development, income inequality and educational expansion (Andersen & Curtis 2012; Breen 2010). Countries with less economic development and high-income countries with large income inequality demonstrate more (self-identified) low social classes (Andersen & Curtis 2012). Moreover, educational expansion can lead to a large group of the upper class (with high volume of cultural and economic capital) (Breen 2010). Because there are transformation processes between all types of capital and because countries’ macro-characteristics influence capital volume and composition, it is hypothesized that there will be a greater proportion of social classes with low general capital volume and deprived capital composition in countries with low economic development (such as Ukraine, Kosovo and Albania), in countries with high income inequality (such as Israel, Southern European and Anglo-Saxon countries) and in countries with low educational expansion (such as Southern European countries). A greater proportion of social classes with high general capital volume and rich capital composition will be found in their counterparts (such as Western and Northern European countries).

b. also **included the actual country-level indicators they** describe in the Introduction and also Discussion section (e.g., country's level of income inequality, educational democratization, gender inequality) into (if possible) the analyses, or in displaying the results (e.g. GINI coefficient, GDP). In this way, their claims in the Discussion section about why such country differences might exist, could be presented more firmly (backed-up with actual data).

Thank you for this suggestion, we have included GDP per capita (in US$), the GINI index and Expenditure on education (% of the GDP), in Europe in 2012 and added these numbers to our hypothesis (see above). These numbers are also added to the results. We have deleted the figure and inserted a Table in which it was easier to include the macro-indicators. The results and discussion have been rewritten to also include the macro-indicators.

|  | **GDP per capita (US$)** | **GINI** | **Educational Expansion^1^** | **Low-educated deprived** | **Middle- educated deprived** | **High Psy/soc/eco capital** | **Upward well-off** | **High Psy/soc/cult capital** | **Generationally well-off** |
| --- | --- | --- | --- | --- | --- | --- | --- | --- | --- |
| **Kosovo** | 3,410 | 29 | *n.a.* | 47 | 8 | 15 | 4 | 25 | 2 |
| **Ukraine** | 4,004 | 24.7 | 6.44 | 10 | 37 | 8 | 0 | 35 | 10 |
| **Albania** | 4,247 | 29 | 3.31 | 70 | 1 | 6 | 15 | 3 | 4 |
| **Bulgaria** | 7,430 | 36 | 3.48 | 35 | 20 | 0 | 2 | 31 | 12 |
| **Hungary** | 12,984 | 30.8 | 4.14 | 24 | 50 | 0 | 0 | 6 | 20 |
| **Poland** | 13,010 | 33 | 4.86 | 35 | 1 | 30 | 24 | 1 | 8 |
| **Lithuania** | 14,367 | 35.1 | 4.76 | 33 | 23 | 0 | 4 | 31 | 9 |
| **Russian Federation** | 15,420 | 40.7 | 3.79 | 23 | 38 | 12 | 0 | 23 | 3 |
| **Estonia** | 17,403 | 32.9 | 4.72 | 17 | 22 | 2 | 5 | 39 | 16 |
| **Slovakia** | 17,498 | 26.1 | 3.86 | 15 | 57 | 0 | 0 | 9 | 18 |
| **Czechia** | 19,870 | 26.1 | 4.22 | 1 | 62 | 0 | 0 | 24 | 13 |
| **Portugal** | 20,563 | 36 | 4.95 | 54 | 0 | 7 | 33 | 0 | 7 |
| **Slovenia** | 22,641 | 25.6 | 5.62 | 17 | 36 | 4 | 0 | 25 | 18 |
| **Spain** | 28,322 | 35.4 | 4.47 | 36 | 1 | 22 | 26 | 0 | 15 |
| **Cyprus** | 28,910 | 34.3 | 5.92 | 42 | 11 | 18 | 22 | 0 | 7 |
| **Israel** | 33,156 | 41.3 | 5.59 | 29 | 14 | 26 | 11 | 9 | 11 |
| **Italy** | 35,051 | 35.2 | 4.06 | 44 | 10 | 27 | 7 | 5 | 8 |
| **France** | 40,870 | 33.1 | 5.46 | 23 | 4 | 51 | 12 | 2 | 9 |
| **United Kingdom** | 42,497 | 33.1 | 5.63 | 22 | 6 | 38 | 20 | 1 | 13 |
| **Germany** | 43,855 | 31.1 | 4.93 | 16 | 0 | 58 | 18 | 2 | 7 |
| **Belgium** | 44,670 | 27.5 | 6.26 | 25 | 13 | 26 | 17 | 1 | 19 |
| **Iceland** | 45,995 | 26.8 | 7.58 | 0 | 17 | 39 | 24 | 6 | 13 |
| **Finland** | 47,708 | 27.1 | 7.15 | 0 | 5 | 39 | 6 | 41 | 9 |
| **Ireland** | 48,943 | 33.2 | 6.16^2^ | 32 | 7 | 37 | 15 | 0 | 8 |
| **Netherlands** | 50,070 | 27.6 | 5.41 | 36 | 7 | 22 | 20 | 0 | 15 |
| **Sweden** | 58,037 | 27.6 | 7.57 | 11 | 15 | 39 | 15 | 7 | 12 |
| **Denmark** | 58,507 | 27.8 | 7.24 | 6 | 2 | 48 | 17 | 10 | 15 |
| **Switzerland** | 85,836 | 31.6 | 4.9 | 10 | 0 | 59 | 20 | 0 | 10 |
| **Norway** | 102,175 | 25.7 | 7.33 | 0 | 10 | 33 | 33 | 6 | 18 |
| ^1^Government expenditure on education, total (% of GDP); ^2^ Data from 2009 (data 2012 unavailable); n.a.: not available | | | | | | | | | |

**Minor comments:**
1. sometimes I miss **references** to claims made in the Introduction section:
- page 3, line 79: PsyCap can .....high-quality life.

Four References have been added to his sentence. The references added are:

PsyCap leads to:

- Good connections (Guo et al. 2020)
- Good jobs (Cenciotti et al. 2017)
- Wealth (Judge & Hurst 2008)
- High quality of life (Santisi et al. 2020)

- page 8, line 204: However, ....grow or decline.

This has been reported in the book “Psychological capital and Beyond” by Luthans et al. (2015). This reference has been added to the manuscript.

2. The authors often use 'etc' after giving examples, which is ambiguous and should be removed (line 104, 177, 220, 251, 254).

In the reported lines with “etc’” in the sentence, we have rewritten the sentences and deleted the “etc”.

3. There is still one claim in the Discussion section that I believe should be formulated more carefully since this cannot be concluded from the results of this cross-sectional study, namely on page 29, line 628-629: This finding also underscores....and vice versa.

We appreciate the alertness of Reviewer 1. The problem with using cross-sectional data is that you cannot make claims about the direction of the relation. With our study, we cannot say that PsyCap leads to social capital or social capital to PsyCap, you can only say that the two are related, in the moment in time under study. Therefore, the sentence has been changed to: This finding also underscores the importance of the association between social capital and PsyCap.

**Reviewer 2:**

Reviewer #2: Thank you for your thorough revisions in response to the reviewer's feedback. While I acknowledge the effort invested, several key issues persist:

1) The justification for introducing the concept and measurement of capital remains unclear.

Thank you for this comment. We hope we understood it correctly and understand that Reviewer 2 is asking for a justification of PsyCap to be included in the Bourdieusian capital framework. Therefore, we have rewritten the second paragraph of our manuscript. This paragraph is now dedicated to the added value of PsyCap into social inequality research (in a concise manner). The added value of PsyCap, is adding a more “personal” element, free from socialization into the drivers of social inequality.

The selection of measurement tools appears arbitrary, for example in excluding **occupation** from capital measures. This raises concerns about the consistency and validity of the measurements and challenges the characterization of these constructs as "capital."

Thank you for this comment. We acknowledge the importance of selecting the correct measurement tools for the different types of capital. The capital types that are included are selected with great care (please see Appendix A, for our answer to comment 1 from Reviewer 2 in the first round of revisions, who made a similar remark).

Firstly, we have deleted the sentence “we only selected “pure” capitals” from our manuscript as it might reflect a sense of superiority. This is not the purpose of the selected measurement tools. We simple want to test our first hypothesis (the transferability of capitals), which requires items that distinctly reflect only one type of capital. We want to stress that for other research purposes occupational status can be a good indicator to construct social classes.

The exclusion of “occupation” has a specific reason. Occupation reflects both cultural and economic capital (that is also the reason why many class schemes rely only on the characteristics of people’s occupation). Someone’s occupation is frequently a direct result of someone’s education and training, while occupation is also frequently used to reflect someone’s level of income (i.e., economic capital). Although our data include measures for individuals’ occupational status and parent’s occupational status, we decided to not use these items. Parents’ and the respondents’ own occupational status is not a “distinct” measure of for instance economic capital as it also entails cultural capital.

By using occupational position, we would be unable to test our first aim (the transferability between different capitals). That is the reason why we used “distinct” economic capital variables, such as perception on income and “distinct” cultural capital variables: own’s and parent’s level of education.

This sentence was added to the methods section:

“To adequately test the transferability between different capitals, we selected measures that distinctly represent only one form of capital. Therefore, items that could account for two capital dimensions are excluded. For instance, occupational status reflects both cultural capital (as educational attainment often is a prerequisite for access to certain professions) and economic capital (as a job is a primary mean of income and financial security) and is subsequently omitted from our analyses.”

2) Similarly, the exclusion of ‘hope’ from the PsyCap construct due to data constraints raises questions about the integrity of the composite. Clarification is needed regarding whether it is appropriate to continue labeling it as PsyCap or if it should be considered a composite of other factors. Furthermore, the potential contributions of this concept and measurement to existing literature are not clearly articulated.

Thank you for pointing our attention to the limitations in the PsyCap construct. We partly agree with Reviewer 2 that we need to be more careful in labelling our construct as PsyCap. Therefore, we have now more clearly indicated in the manuscript that we measure “a proxy” of PsyCap.

In large cross-national surveys such as the ESS one often needs to work with proxy variables. For PsyCap this means that we have adequate items for self-efficacy, resilience and optimism, but not for hope. What we did in our revised paper, however, is to explicitly mention that our measure of PsyCap is a proxy measure.

The consequence of using a proxy is one of construct validity. We, however, feel that this issue is not too severe because (a) PsyCap is a higher-order construct that captures shared variance between four lower-order constructs. Because three of those constructs are well represented in our proxy measure and because our analyses show that they clearly load on one factor and are internally consistent, we believe that our proxy captures this common core rather well.

Moreover, (b) Hope consists of three things: agency, pathways, and goal (Snyder & Lopez 2002). Whereas pathways and goals are not well represented in the items, agency is to some extent reflected in the resilience item "How difficult or easy do you find it to deal with important problems that come up in your life". In addition, according to Huppert et al. (2013), optimism also refers to hopefulness, by reflecting its emotional facet. So, hope is somewhat captured in the way optimism and resilience are measured.

We added this issue to our limitations. We added several sentences in the limitations section:

“Additionally, the indicator for PsyCap is only a proxy for the underlying theoretical concept and the measurement of the dimension hope was suboptimal. Yet, because (1) PsyCap is a second-order construct, (2) the subscales clearly load on one factor and are internally consistent and (3) hope is somewhat captured in the way optimism and resilience are measured, the inadequate measurement of one indicator should not be problematic. In future research, it would be highly desirable to include more and other measures for the capitals to validate our research results.”

Moreover, we have explained the construction of the proxy of PsyCap better in the data section:

“Although in the original concept of PsyCap the dimension of hope is represented; in the ESS6, no direct measure for hope was included [26]. However, PsyCap is a higher-order construct that captures shared variance between the four lower-order constructs [28]. Because three of those constructs are well represented in our proxy measure and because our analyses show that they clearly load on one factor and are internally consistent (see below [in the manuscript]), we believe that our proxy measure captures this common core rather well. Finally, hope consists of three things: agency, pathways, and goal [28]. Whereas pathways and goals are not well represented in our proxy measure, agency is to some extent reflected in the item "How difficult or easy do you find it to deal with important problems that come up in your life". In addition, optimism – included in the ESS6 – refers partly to hopefulness (namely the emotional facet) [27]. Because of these reasons, we are confident in the construct validity of our PsyCap proxy measure.”

Finally, the potential contribution of PsyCap to the Bourdieusian literature has been better explained in the introduction of the paper (see comment 1 of Reviewer 2). Adding PsyCap to the analysis of social inequality contributes to both the sociological and psychological literature (which is explained in the discussion section).

3) Additionally, the discussion surrounding the number of Latent Class Analysis (LCA) clusters lacks clarity -- which I have missed in my first-round review. While a 6-cluster model is presented, the marginal decrease in Bayesian Information Criterion (BIC) raises questions about the meaningfulness of these clusters (0.11%). The difference in BIC between 1-cluster and 2-clusters is also negligible (3%), suggesting a lack of significance. It is imperative that the authors provide a robust defense for the meaningfulness of these clusters, tying back to the broader question of the necessity of PsyCap.

Thank you for pointing our attention to the unclarity regarding the selection of number of social classes. In the revised paper we elaborate on the selection of the classes based on the following arguments (in the methods section):

For selecting the final cluster model and subsequently the number of social classes, two criteria were taken into consideration: (1) the best-fitting model and (2) the theoretical meaning of the models. Regarding the former, the statistically best-fitting model was determined by examining changes in formal indicators of model fit between different cluster solutions. These criteria – the Akaike Information Criterion (AIC), Bayesian Information Criterion (BIC) and Consistent Akaike Information Criterion (CAIC) – help to find the most parsimonious model (Van Aerden 2018). They show how accurately the estimated model represents the pattern of relations observed in the data: the lower their values, the better the model fits the data. When the extent of improvement in the model fit indices shows a clear drop after the inclusion of an extra cluster, this suggests saturation. In the case of two solutions with a relatively similar fit, the model with fewer latent classes is preferred over the model offering more, but smaller clusters.

Yet this considerable “drop” should not be interpreted with regards to the original BIC value (432857.6). So, the drops that need to be compared are in column 5 of Table 4 of the manuscript (see, **Δ**BIC). We should be comparing the number 13,980 to 2,642 (19%) which is a considerable drop. Afterwards, the drops are getting smaller, but are still considered drops. The considerable drop can be seen for the 7-cluster model (as for the 8-cluster model there is no more drop -126.3).

**Table 4. Comparison of selected fit indices and degree of model improvement over the different cluster models, ESS6, age 21-65, 29 countries, weighted (N=35,489)**

| **Model** | **BIC** | **AIC** | **CAIC** | **ΔBIC** | **ΔAIC** | **Δ CAIC** |
| --- | --- | --- | --- | --- | --- | --- |
| 1 cluster | 432857.6 | 431331.8 | 433037.6 |  |  |  |
| 2 clusters | 418877.4 | 417054.9 | 419092.4 | 13980.2 | 14276.9 | 13945.2 |
| 3 clusters | 416234.9 | 414115.6 | 416484.9 | 2642.6 | 2939.3 | 2607.6 |
| 4 clusters | 414817.8 | 412401.8 | 415102.8 | 1417.1 | 1713.8 | 1382.1 |
| 5 clusters | 413785.5 | 411072.8 | 414105.5 | 1032.3 | 1329.0 | 997.3 |
| 6 clusters | 413312.9 | 410303.5 | 413667.9 | 472.6 | 769.3 | 437.6 |
| 7 clusters | 413054.1 | 409748.1 | 413444.1 | 258.7 | 555.4 | 223.7 |
| 8 clusters | 413180.5 | 409577.7 | 413605.5 | -126.3 | 170.4 | -161.3 |

Obviously, the fit of the measurement model is a very important element in evaluating the different cluster solutions. Nevertheless, this is not the only criterion that has to be taken into account. Because any measurement model should have theoretical meaning, the substantive interpretation of the obtained clusters is a second important element (Van Aerden 2018).

Therefore, in order to decide on the final number of clusters to be retained, the relations between the latent classes and capital-indicators (the conditional probabilities) are examined for the three statistically most acceptable models, to assess the meaningfulness of each extra cluster.

We interpreted a 5-, 6- and 7-class solution. We were able to make the most sensible interpretation using the 6-class solution. When comparing the 5-class to the 6-class solution, the 6-class solution adds a profoundly different profile. Yet, when comparing the 6-class to the 7-class solution, the 7-class solution adds a profile with a very small cluster size (6%) and no profoundly different profile. Based on this information, we decided that the 6-class model best represents reality.

We also changed one sentence in our results section to clarify the selection of the 7-cluster model as the best-fitting model:

Going up to a 7-cluster model showed a decrease ***in change*** in BIC, AIC, and CAIC.

**Changes to references in the manuscript**

**Please review your reference** list to ensure that it is complete and correct.

If you have cited papers that have been retracted, please include the rationale for doing so in the manuscript text, or remove these references and replace them with relevant current references. **Any changes to the reference list should be mentioned in the rebuttal letter that accompanies your revised manuscript**. If you need to cite a retracted article, indicate the article’s retracted status in the References list and also include a citation and full reference for the retraction notice.

Several references were added in the introduction (as asked by Reviewer 1):

Santisi, G., Lodi, E., Magnano, P., Zarbo, R., & Zammitti, A. (2020). Relationship between psychological capital and quality of life: The role of courage. *Sustainability*, *12*(13), 1–14. https://doi.org/10.3390/su12135238

Guo LX, Liu CF, Yain YS. Social Entrepreneur’s Psychological Capital, Political Skills, Social Networks and New Venture Performance. Front Psychol. 2020;11: 1–10. doi:10.3389/fpsyg.2020.00925

Cenciotti R, Alessandri G, Borgogni L. Psychological Capital and Career Success Over Time: The Mediating Role of Job Crafting. J Leadersh Organ Stud. 2017;24: 372–384. doi:10.1177/1548051816680558

Judge TA, Hurst C. How the rich (and happy) get richer (and happier): Relationship of core self-evaluations to trajectories in attaining work success. J Appl Psychol. 2008;93: 849–863.

Because new information was added into the statistical section, we also added the following references:

World Bank. https://data.worldbank.org/. 2024.

Magidson J, Vermunt JK. Latent class models. In: Kaplan D, editor. The Sage Handbook of Quantitative Methodology for the Social Sciences. Thousand Oaks: Sage Publications.; 2004. pp. 175–198.

Added: to explain “hope”:

Snyder, C. R., & Lopez, S. J. (2002). *Handbook of Positive Psychology* (C. R. Snyder & S. J. Lopez (eds.)). Oxford University Press.

**References**

Andersen, R., & Curtis, J. (2012). The polarizing effect of economic inequality on class identification: Evidence from 44 countries. *Research in Social Stratification and Mobility*, *30*(1), 129–141.

Avey, J. B., Reichard, R. J., Luthans, F., & Mhatre, K. H. (2011). Antecedents and Outcomes of Workplace Incivility. *Human Resources Development Quarterly*, *22*(2), 127–152. https://doi.org/10.1002/hrdq

Bourdieu, P. (2021). *Forms of Capital. General Sociology, Volume 3. Lectures at the collège de France, 1983-1984* (P. Champagne, J. Duval, F. Poupeau, & M.-C. Rivière (eds.)). Polity.

Breen, R. (2010). Educational expansion and social mobility in the 20th Century. *Social Forces*, *89*(2), 365–388. https://doi.org/10.1353/sof.2010.0076

Carmo, R. M., & Nunes, N. (2013). Class and Social Capital in Europe: A transnational analysis of the European Social Survey. *European Societies*, *15*(3), 373–387. https://doi.org/10.1080/14616696.2012.691171

Cenciotti, R., Alessandri, G., & Borgogni, L. (2017). Psychological Capital and Career Success Over Time: The Mediating Role of Job Crafting. *Journal of Leadership and Organizational Studies*, *24*(3), 372–384. https://doi.org/10.1177/1548051816680558

Costa, A. F. da, Machado, F. L., & Almeida, J. F. de. (2009). Social classes and educational assets: A transnational analysis. In *Knowledge and Society, Vol. II: Portugal in the European Context* (pp. 5–20). CIES, ISCTE-IUL/Celta Editora.

Granovetter, M. S. (1973). The Strength of Weak Ties. *American Journal of Sociology*, *78*(6), 1360–1380.

Guo, L. X., Liu, C. F., & Yain, Y. S. (2020). Social Entrepreneur’s Psychological Capital, Political Skills, Social Networks and New Venture Performance. *Frontiers in Psychology*, *11*(June), 1–10. https://doi.org/10.3389/fpsyg.2020.00925

Judge, T. A., & Hurst, C. (2008). How the rich (and happy) get richer (and happier): Relationship of core self-evaluations to trajectories in attaining work success. *Journal of Applied Psychology*, *93*(4), 849–863.

Luthans, F., Youssef-Morgan, C. M., & Avolio, B. J. (2015). *Psychological Capital and Beyond*. Oxford University Press.

Martin, N. D. (2009). Quantifying Social Class: A Latent Cluster Approach. In K. Robson & C. Sanders (Eds.), *Quantifying Theory: Pierre Bourdieu* (pp. 161–173). Springer.

Santisi, G., Lodi, E., Magnano, P., Zarbo, R., & Zammitti, A. (2020). Relationship between psychological capital and quality of life: The role of courage. *Sustainability*, *12*(13), 1–14. https://doi.org/10.3390/su12135238

Snyder, C. R., & Lopez, S. J. (2002). *Handbook of Positive Psychology*. Oxford University Press.

Van Aerden, K. (2018). *Contemporary employment arrangements in Europe and their relation with workers’ well-being.* 311.

**Appendix A**

[Authors]: Thank you for highlighting the valid concern regarding the limited measurement tools for different capitals. Exploring alternative methods is indeed crucial to ensure the robustness of our findings. In the revised manuscript, we included a thoughtful discussion on potential alternative measurement approaches to enhance the validity of our research results.

1) Researchers (Carmo & Nunes 2013; Martin 2009) have developed **measurement items to operationalize the 3 Bourdieusian capitals**. Below a summary of measurement items for Bourdieu's key capitals is given:

1. **Economic Capital:**
   - Income and wealth: Measurement items may include questions about the individual's income, assets, and financial resources.
   - Material possessions: Assessing the ownership of valuable goods, property, and other assets.
2. **Cultural Capital:**
   1) Cultural capital can be **embodied**: These are tastes, preferences, and certain kinds of (practical) knowledge (e.g., assessing familiarity with and appreciation for high culture, such as literature, art, and classical music, but also examining language proficiency, vocabulary, and verbal communication skills);
   2) Cultural capital can be **objectified:** such as books, paintings, machines, and instruments;
   3) Cultural capital can be **institutionalized**, such as titles and diplomas (e.g., measuring the level of formal education completed) (Bourdieu 2021).
3. **Social Capital:**
   - Social networks: Measuring the size and quality of an individual's social connections;
   - Civic participation: Assessing involvement in social groups, clubs, and community activities;
   - Trust: Examining the degree to which individuals trust and are trusted by others (Carmo & Nunes 2013).

Yet, it is not easy to measure each of these dimensions directly using the appropriate data and indicators. For example, it is very difficult to measure the way people organize and produce their social networks. This requires specific data and methodologies that are not available in surveys like the European Social Survey (Carmo & Nunes 2013). Although it is of course interesting to try to measure a theoretical concept in its pure form (and hence develop better measurements) in the end, our manuscript simply tries to show that even with rough proxies we find interesting relationships. Follow-up research that is informed by a capital-approach will focus on transformations between different forms of capital (that is the real contribution of Bourdieu: the idea that some forms of capital can be transformed in others). Such research will be more focused and hence more likely to include better measures. The potential alternative measurement approaches have now been included to our limitations section in the discussion.

3) For sake of clarity we also explain, why we selected the measurements items of our study:

For defining our study’s measurement approaches we found inspiration in the book “Quantifying Theory: Pierre Bourdieu” by Robson and Sanders (eds.) published in 2009. This book and particularly Chapter 13 “Quantifying Social Class: A Latent Clustering Approach” by Nathan D. Martin, together with 2 peer-reviewed papers examining social class in the ESS (Carmo & Nunes 2013; Costa et al. 2009) offered the base for the selected measurement items used in our study.

Nathan D. Martin (2009) measured cultural and economic capital with parent’s education, parent’s income, business ownership of the parent’s, and respondent’s household income. Carmo & Nunes (2013) measured cultural and economic capital using socio-professional indicators (industrial workers, routine employees, self-employed workers, professionals and managers, private professionals and employers and executives). They measured social capital using trust (broken down into personal and institutional trust), sociality practices (measured by informal sociality and participation in social activities) and associational membership (divided into membership of trade unions or professional associations and membership of political parties).

Although our data include measures for individuals’ occupational status, parent’s occupational status, trust and civic participation, we decided to not use some of these items. Firstly, parent’s and own’s occupational status is not a “pure” measure of for instance economic capital as it also entails cultural capital. By using these measures, we would be unable to test our first aim (the transferability between different capitals). Instead, we used “pure” economic capital variables, such as perception on income and “pure” cultural capital variables: own’s and parent’s level of education. Secondly, we also limited the selected measures of capital. For our study, we decided to focus on maximum two specific measurement scales (e.g. weak ties and strong ties (Granovetter 1973)) from the social capital dimension within ESS6) that focus on social capital itself rather than its outcomes (i.e., social trust). We limited the number of measurement items for each capital dimension to maximum two to balance the Latent Class Analysis and presumably simplify the analysis while still capturing the essence of each capital type.
