# Supplementary material for: Psychological capital and social class: A capital approach to understanding positive psychological states and their role in explaining social inequalities
Source: PLoS One. 2024 Sep 9;19(9):e0310031. doi: 10.1371/journal.pone.0310031 (PMC11383244; doi:10.1371/journal.pone.0310031)
Supplement: Attachment — Submitted filename: Response to reviewers.docx [file pone.0310031.s003.docx]

Dear Editor,

Dear reviewers,

We are pleased to hear that the reviewers and you found that our revised submission greatly improved. We appreciate the recommendation for minor revisions.

In this document, we address the reviewer’s comments, which are marked in **bold**, and our explanation follows right after. Where appropriate, we also include excerpts from the revised manuscript in text boxes.

Lastly, we reviewed our reference list to ensure that it is complete and correct.

Thank you for taking the time to provide detailed comments and suggestions. We believe that the suggested changes have shaped and strengthened our manuscript.

Yours sincerely,

The Authors

**Reviewer #2:**

**[Reviewer #2]: I appreciate the extensive revisions you have made and the effort to address the concerns regarding your measurement of PsyCap. Your detailed response highlights your commitment to improving the clarity and robustness of your study.**

[Authors:] We are grateful that Reviewer 2 sees the efforts we made to improve our study. Below, we address the final comment.

**[Reviewer #2]: One thing I would like to highlight is the PsyCap measurement. I don't think the PsyCap measurement should be justified as stated in the discussion: "because (1) PsyCap is a second-order construct, (2) the subscales clearly load on one factor and are internally consistent, and (3) hope is somewhat captured in the way optimism and resilience are measured, the inadequate measurement of one indicator should not be problematic." (p.32).

This justification overlooks the importance of each component in maintaining the integrity of the PsyCap construct (see the discussion of similarities and differences among four dimensions of PsyCap in Lutherans & Youssef-Morgan 2017). Each of the elements contributes uniquely to the overall construct, and their interrelationships do not justify the exclusion of any single component. The integrity and theoretical foundation of PsyCap rest on the presence of all four factors, and omitting one undermines the validity of the construct.**

**The impact of not fully measuring PsyCap could potentially skew the study's findings and their implications. Although I understand the limitation of secondary data, this needs to be explicitly stated in the manuscript. If possible, I suggest adding a discussion of the expected outcomes that might result from the inclusion of the hope dimension.**

**For instance, in relation to one of the main findings—that countries where governments allocate large funds to education systems (like Norway, Finland, and Denmark) have classes with lower prevalence of low cultural capital—each social and cultural context can provide different levels of hope for individuals.**

**This variation in hope levels could potentially alter the latent classes and demonstrate different cross-cultural dynamics. By including hope, you could offer a more comprehensive analysis of how different components of PsyCap interact with educational and cultural policies across diverse contexts — for example.**

[Authors:] Thank you for pointing our attention to the paper of Luthans & Youssef-Morgan [1] in the Annual Review of Organisational Psychology and Organisational Behavior. After careful reading of this paper, we altered our discussion. We now make an in-depth discussion of how our results can be skewed due to the lack of a direct measure of “hope” in our PsyCap construct. Moreover, we have deleted our original justification that Reviewer 2 mentions in their review, both in the discussion and in the methods section.

We added the following sentences to our discussion:

The lack of “hope” in our PsyCap construct can potentially skew our results as PsyCap is a second-order construct based on shared commonalities of first-order psychological resources (hope, resilience, efficacy, and optimism), but also based on their unique characteristics [1]. The discriminant validity of these constructs has been empirically established [2]. Unfortunately, the ESS data do not include a proxy for the ‘hope’ element of PsyCap. Given that we used PysCap to define clusters, our analysis may have missed a social class cluster distinctly characterized by waypower, which is unique to hope. This “overlooked” social class might also have a specific cross-country distribution as the level of hope varies between countries and may be related to policies like government spending on education and social services [3]. Whether such a cluster exists and how it is distributed over countries is an empirical question that we cannot answer with the available data. There are, however, certain indications that suggest that the component “hope” does not distinctly dominate profiles within populations. Indeed, studies based on person-centered latent profile analyses of the PsyCap components show a gradient in the profiles (ranging from low, to moderate to rich (general) PsyCap) [4–6] or in other cases the other PsyCap components (optimism or resilience) seem to dominate the profiles [7,8]. Our research shows that there is much to be gained by including PsyCap in comparative research and recommends that a more comprehensive measure for PsyCap is included in large-scale survey projects like the European Social Survey.

**References**

1.	Luthans F, Youssef-Morgan CM. Psychological Capital: An Evidence-Based Positive Approach. Annu Rev Organ Psychol Organ Behav. 2017;4: 339–366. doi:10.1146/annurev-orgpsych-032516-113324

2.	Luthans F, Youssef CM. Emerging Positive Organizational Behavior. J Manage. 2007;33: 321–349. doi:10.1177/0149206307300814

3.	Krafft AM, Guse T, Slezackova A, editors. Hope across cultures: Lessons from the International Hope Barometer. Cham: Springer International Publishing; 2023. doi:10.1007/978-3-031-24412-4

4.	Song L, Zhou Y, Zheng J, Wang Y, Li H, Feng X. Psychological capital of Chinese employees: Investigating its measurement and latent profiles. J Pacific Rim Psychol. 2024;18. doi:10.1177/18344909241254497

5.	Gao Y, Yue Y, Li X. The relationship between psychological capital and work engagement of kindergarten teachers: A latent profile analysis. Front Psychol. 2023;14. doi:10.3389/fpsyg.2023.1084836

6.	Teng M, Wang J, Jin M, Yuan Z, He H, Wang S, et al. Psychological capital among clinical nurses: A latent profile analysis. Int Nurs Rev. 2023. doi:10.1111/inr.12918

7.	Bouckenooghe D, De Clercq D, Raja U. A person-centered, latent profile analysis of psychological capital. Aust J Manag. 2019;44: 91–108. doi:10.1177/0312896218775153

8.	Geremias RL, Lopes MP, Soares AE. Psychological Capital Profiles and Their Relationship With Internal Learning in Teams of Undergraduate Students. Front Psychol. 2022;13. doi:10.3389/fpsyg.2022.776839

9.	Platania S, Paolillo A. Validation and measurement invariance of the Compound PsyCap Scale (CPC-12): a short universal measure of psychological capital. An Psicol. 2022;38: 63–75. doi:10.6018/analesps.449651

10.	Luthans F, Youssef-Morgan CM, Avolio BJ. Psychological Capital and Beyond. Oxford University Press; 2015.
